# Disentangling the Evolutionary History of Feo, the Major Ferrous Iron Transport System in Bacteria

**DOI:** 10.1128/mbio.03512-21

**Published:** 2022-01-11

**Authors:** Camilo Gómez-Garzón, Jeffrey E. Barrick, Shelley M. Payne

**Affiliations:** a Department of Molecular Biosciences, University of Texas at Austin, Austin, Texas, USA; University of Hawaii at Manoa

**Keywords:** bacteria, bioinformatics, evolution, phylogenetics, *Vibrio cholerae*, Feo, bacterial evolution, iron transport

## Abstract

Iron acquisition is essential for almost all living organisms. In certain environments, ferrous iron is the most prevalent form of this element. Feo is the most widespread system for ferrous iron uptake in bacteria and is critical for virulence in some species. The canonical architecture of Feo consists of a large transmembrane nucleoside triphosphatase (NTPase) protein, FeoB, and two accessory cytoplasmic proteins, FeoA and FeoC. The role of the latter components and the mechanism by which Feo orchestrates iron transport are unclear. In this study, we conducted a comparative analysis of Feo protein sequences to gain insight into the evolutionary history of this transporter. We identified instances of how horizontal gene transfer contributed to the evolution of Feo. Also, we found that FeoC, while absent in most lineages, is largely present in the *Gammaproteobacteria* group, although its sequence is poorly conserved. We propose that FeoC, which may couple FeoB NTPase activity with pore opening, was an ancestral element that has been dispensed with through mutations in FeoA and FeoB in some lineages. We provide experimental evidence supporting this hypothesis by isolating and characterizing FeoC-independent mutants of the Vibrio cholerae Feo system. Also, we confirmed that the closely related species Shewanella oneidensis does not require FeoC; thus, *Vibrio* FeoC sequences may resemble transitional forms on an evolutionary pathway toward FeoC-independent transporters. Finally, by combining data from our bioinformatic analyses with this experimental evidence, we propose an evolutionary model for the Feo system in bacteria.

## INTRODUCTION

Feo is the most widespread system for ferrous iron (Fe^2+^) transport in bacteria (1, 2). This system is critical for growth in some species, especially in environments where Fe^2+^ is the prevalent form of iron. For instance, environmental bacteria such as Bradyrhizobium japonicum, a nitrogen-fixing plant symbiont, and Shewanella oneidensis rely on Feo-mediated iron uptake (3, 4). Likewise, Fe^2+^ transport by Feo can determine the outcomes of bacterial infections within animal and plant hosts in some pathogens (2, 5). These include the human intestinal and gastric pathogens Salmonella enterica (6) and Helicobacter pylori (7) and plant pathogens such as *Xanthomonas* species (8, 9). Despite its importance in bacterial iron acquisition, the evolution and working mechanism of the Feo system remain poorly understood.

The first characterized Feo system (in Escherichia coli) comprises three proteins, FeoA, FeoB, and FeoC, encoded in an operon. However, the latter component, FeoC, is present in only a few species, primarily in the *Gammaproteobacteria* group (2). These proteins work together to transport Fe^2+^ by assembling a multimeric complex embedded in the cytoplasmic membrane (10). FeoA and FeoB are the structural elements of the complex. FeoB is a transmembrane protein of approximately 80 kDa that likely forms a trimeric pore. Its cytoplasmic N-terminal domain (NFeoB) shares homology with eukaryotic G proteins and hydrolyzes GTP and/or ATP, depending on the species. FeoA is a small cytoplasmic protein (<10 kDa) that interacts with FeoB and is required for complex formation (10, 11), but little is known about its function. The role of FeoC, also a small cytoplasmic protein, is even more elusive. FeoC is not widespread among bacteria, and its sequence is poorly conserved (1). This protein is thought to be a regulator of Feo-mediated iron uptake, but its mechanism remains unknown.

Previously, our laboratory has used the human pathogen Vibrio cholerae as a model organism for studying the Feo system. We have determined that FeoC interacts with NFeoB (11) and that the three proteins in the Feo complex assemble in a 1:1:1 stochiometric ratio (12). FeoC is not required for complex formation, but complexes formed in its absence are not functional (10). Therefore, FeoC is an essential component of the V. cholerae Feo system.

Several potential roles for FeoC have been proposed, including that it acts as a transcription factor (13), a protein stabilizer (14, 15), an iron sensor (16), or a GTPase accessory protein (1). Nonetheless, these roles are hypothetical or have not been experimentally tested in more than one species. Indeed, due to the low levels of sequence conservation in FeoC sequences, most of these putative functions do not seem to be feasible in all bacterial species. That is, those motifs considered critical for the FeoC function proposed in one species are often absent in the FeoC sequences of other species. Thus, studying the evolutionary history and phylogenetic distribution of FeoC might provide insight into the actual role and working mechanism of this protein.

In this study, we examined the phylogenetics and gene architecture of the Feo system in bacteria. Specifically, we analyzed the evolutionary history of Feo, focusing on the distribution of the accessory proteins FeoA and FeoC. Since FeoC is the least-conserved element of this system, although it is prevalent in *Gammaproteobacteria*, we complemented our bioinformatic analyses with experiments that tested whether the requirement for this protein could be bypassed. Our findings are consistent with the hypothesis that the ancestral version of the Feo operon included an FeoC element, but most bacterial lineages have dispensed with this protein by accumulating mutations in FeoA and FeoB.

## RESULTS

### FeoC, unlike FeoA and FeoB, is not widespread among bacterial species.

**FIG 1:**
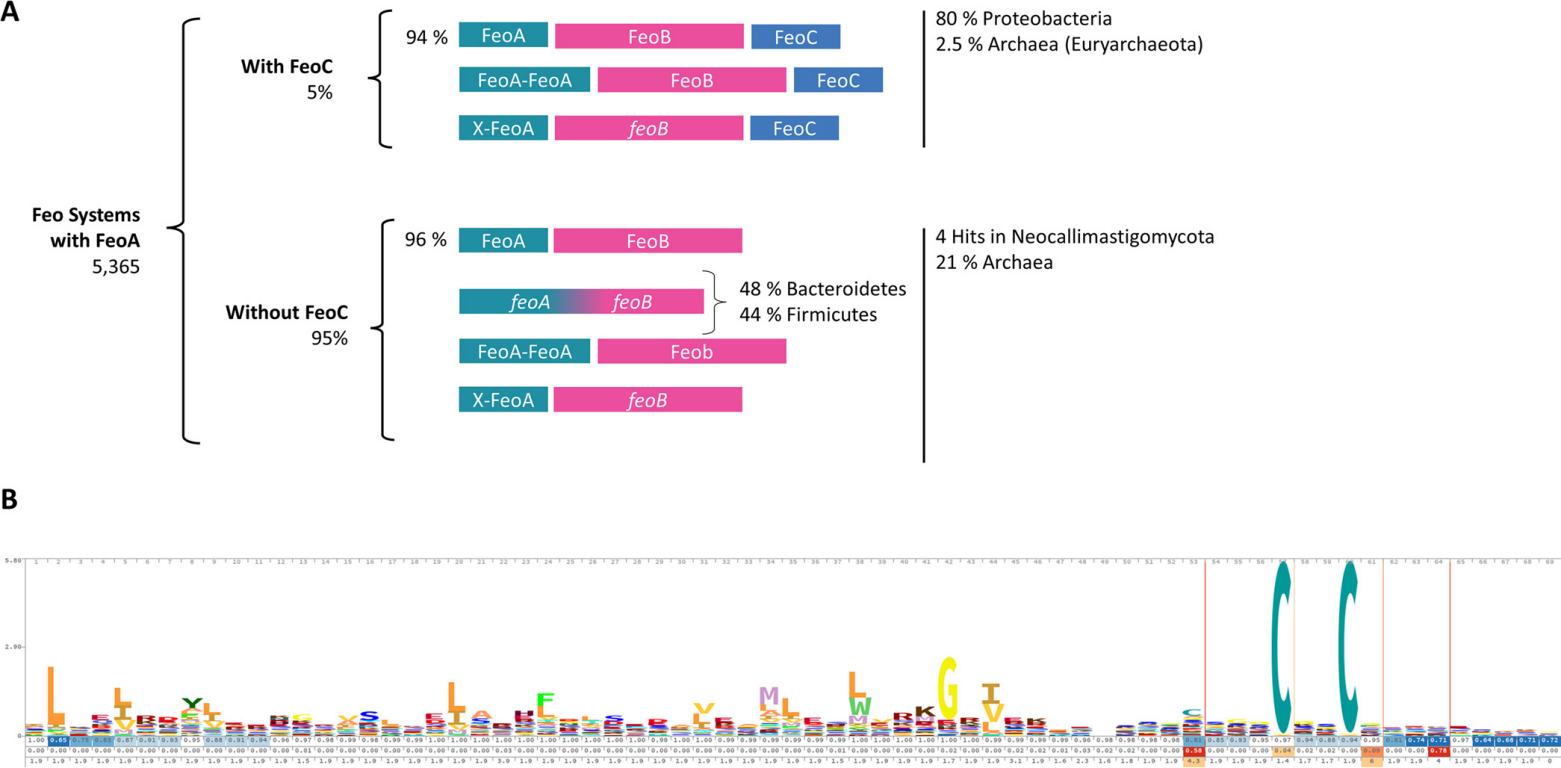
Distribution of the Feo architectures deposited in the UniProt Reference Proteomes database. (A) Graphic summary of the results for Feo systems obtained using the HMM Search pipeline through the HMMER Web server. The HMM profile of FeoA was used as a query to identify Feo elements in bacterial proteomes. Next, the architecture of the retrieved systems was classified using the HMM profiles of FeoB and FeoC. (B) Sequence logo representation of the FeoC family HMM profile based on the 523 sequences deposited in Pfam (PF09012).

Since FeoC is the least-conserved protein of the Feo system, its phylogenetic distribution and how its sequence varies among species may inform us about its role and origin. To this end, we sought to identify FeoC orthologs in reference databases; however, a BLASTP search failed in this task due to the poor conservation of FeoC amino acid sequences. For instance, a BLASTP search of E. coli FeoC against the V. cholerae genome did not yield any matches, and neither did the search of V. cholerae FeoC against the E. coli genome. This means that the FeoC proteins from these species cannot be classified as orthologs using reciprocal BLASTP, which is the default method for ortholog prediction in bacteria. In light of this result, we carried out new searches using HMMER (17). We used the FeoA and FeoC hidden Markov model (HMM) profiles deposited in Pfam as queries to search the UniProt Reference Proteomes database. With this approach, we found 5,365 putative Feo systems, 95% of which contain only FeoA and FeoB elements (Fig. 1A). The small number of FeoC-containing systems are unevenly distributed, with most of them belonging to the *Gammaproteobacteria* and 2.5% belonging to the archaeal phylum *Euryarchaeota*. When using the FeoA HMM profile as a query, we found four hits within the fungal phylum Neocallimastigomycota. A further examination of these matches showed that they either correspond to a protein with both FeoA- and FeoB-like domains or are annotated upstream of an *feoB*-like gene (see Tables S1 and S2 at https://doi.org/10.6084/m9.figshare.17082758).

The distribution of Feo proteins that we found resembles that reported in previous studies (2, 13) and is consistent with the global distribution of Feo-associated protein domains in different databases (see Tables S3 and S4 at the URL mentioned above), indicating that this finding is not due to sampling bias produced by the specific queries and database that we used.

All the Feo operons that we identified in bacteria have an *feoB* gene and at least one copy of *feoA* or an FeoA-like domain fused to the N terminus of FeoB. We also found instances in which FeoA has an additional domain or two FeoA-like domains are combined in a single protein (Fig. 1A). We did not find any system containing FeoC and FeoB but not FeoA. Altogether, these results show that FeoA and FeoB are highly conserved as essential components of the Feo system, while FeoC is present in only a few species. In all the species containing FeoC, *feoC* is downstream of *feoAB*, in the same direction and with similar intergenic spacing.

Next, we explored the sequence conservation of FeoC. The FeoC family in Pfam comprises 523 sequences, and its HMM profile was built based on a seed alignment of 15 sequences from diverse bacterial phyla, all annotated as either FeoC domain-containing proteins or FeoC-like transcriptional regulators. The sequence logo representation of this protein family shows that only a few amino acids appear to be conserved (Fig. 1B). Namely, a C-terminal cysteine-rich motif (Cx_2–7_C) between positions 57 and 60 stands out as the most conserved motif, and it is embedded in a region with several insertions ranging from 2 to 5 amino acids (aa).

### FeoC is unevenly distributed in prokaryotes.

Two possible scenarios may explain the limited and uneven presence of FeoC. First, FeoC could be an element recently acquired in some lineages that has been stabilized by positive selection. Alternatively, the ancestral form of the Feo system may have had an FeoC-like component that has been lost in most clades. To evaluate each of these hypotheses, we examined how FeoA, FeoB, and FeoC are distributed across different groups of organisms and whether specific features of FeoA or FeoB correlate with the absence of FeoC.

**FIG 2:**
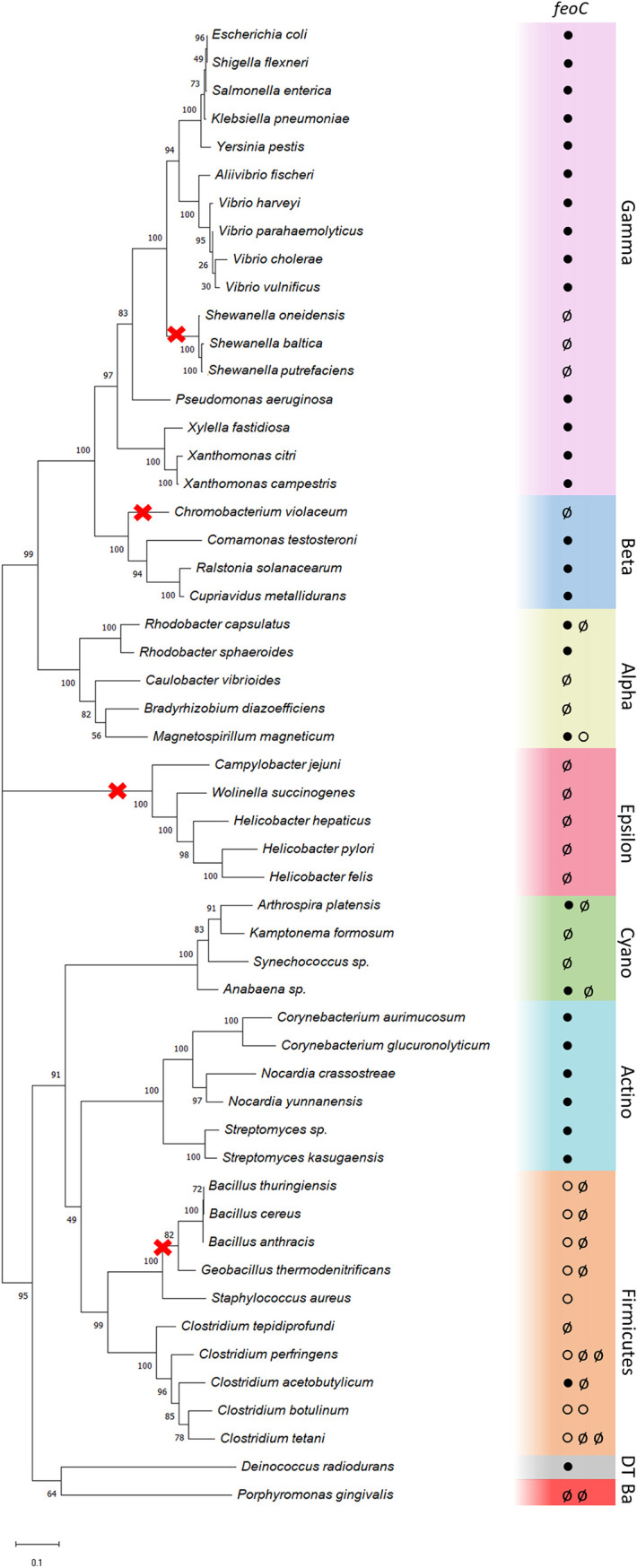
Phylogenetic distribution of FeoC in bacteria. The organismal tree shows the presence/absence of FeoC-like elements in representative bacterial genomes. Colors indicate phylogenetic groups, either phyla (*Actinobacteria* [Actino], *Cyanobacteria* [Cyano], *Firmicutes*, *Deinococcus-Thermus* [DT], and *Bacteroidetes* [Ba]) or proteobacterial classes (*Alpha*-, *Beta*-, *Gamma*-, and *Epsilonproteobacteria*). Circles inside the colored boxes represent the *feoC* status found in each of the Feo loci identified in the corresponding genome: *feoC* annotated (●), no *feoC* identified (ø), or FeoB-associated Cys-rich protein annotated downstream of *feoB* (○). Multiple circles indicate multiple Feo systems in the same genome. The red crosses show potential instances of *feoC* loss events consistent with the evolutionary model that we propose. This tree was constructed using RpoB sequences annotated in each genome by the maximum likelihood method with 300 bootstrap replicates (values over 50% are shown on each node).

We used RpoB sequences from representative genomes to construct an organismal phylogeny of bacterial species with annotated Feo systems. The resulting tree (Fig. 2) reflected the current phylogenetic classification of the selected species. Next, we determined the gene architecture of the Feo operon in each of these species. According to our tree, FeoC is unevenly distributed among clades; therefore, a potential acquisition event of FeoC cannot be assigned to a single node. It is noteworthy that some species lacking *feoC* have a small “FeoB-associated Cys-rich membrane protein” (PF12669 in Pfam) annotated downstream of *feoB*. This oligopeptide is predominant in *Firmicutes* and *Bacteroidetes*, and in some species, it is present as a C-terminal domain of FeoB (see Fig. S1 at https://doi.org/10.6084/m9.figshare.17082773). It contains a cysteine-rich motif (Cx_3–5_Cx_2–3_Cx_6–8_C) commonly associated with iron binding (16).

FeoC proteins from distant species are likely homologs since they can be gathered by a single HMM profile and are located downstream of an *feoB* gene. Yet our reconstruction does not show a single potential origin of FeoC (Fig. 2). While the gene acquisition model cannot be ruled out, the most parsimonious scenario given our results relies on separate events of gene loss. In this regard, our tree shows four potential instances of *feoC* loss: Chromobacterium violaceum, which may have lost *feoC* after diverging from the other *Betaproteobacteria*; the *Epsilonproteobacteria* phylum, which might have a common ancestor without *feoC*; Gram-positive bacilli, which have two Feo gene clusters, one with and one without *feoC*; and the *Shewanella* group, which we analyze further below. In contrast, the gene acquisition model would require multiple instances of horizontal gene transfer (HGT), some of them followed by gene loss events. For example, this would be the case for *Shewanella* species if the common ancestor of the *Proteobacteria* group acquired *feoC* via HGT.

### Horizontal gene transfer has played a role in the evolution of the Feo system.

**FIG 3:**
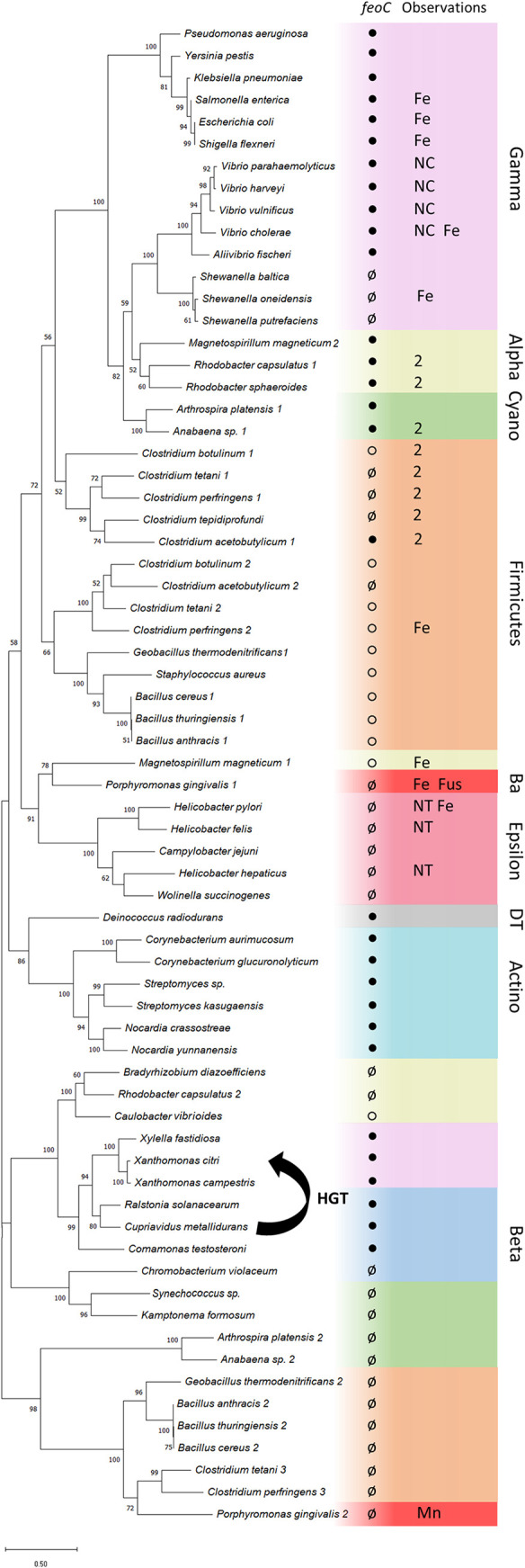
Evolutionary history of the Feo system. This tree was constructed by the maximum likelihood method based on the alignment of concatenated Feo protein sequences from each locus. Bootstrap values were calculated with 300 replicates, and those values over 50% are shown on the corresponding nodes. Colors indicate phylogenetic groups, either phyla (*Actinobacteria* [Actino], *Cyanobacteria* [Cyano], *Firmicutes*, *Deinococcus-Thermus* [DT], and *Bacteroidetes* [Ba]) or proteobacterial classes (*Alpha*-, *Beta*-, *Gamma*-, and *Epsilonproteobacteria*). Circles inside the colored boxes represent the *feoC* status as annotated for the corresponding locus: *feoC* annotated (●), no *feoC* identified (ø), or FeoB-associated Cys-rich protein annotated downstream of *feoB* (○). Some additional observations are also shown: FeoA and FeoB domains fused as a single protein (Fus), two *feoA* genes annotated in the same locus (2), experimentally verified activity toward ferrous iron (Fe) or manganese(II) (Mn), FeoC lacking cysteine residues (NC), and *feoA* and *feoB* not encoded together at the same genomic location (NT). The horizontal gene transfer (HGT) event that we propose between the beta- and gammaproteobacterial groups is shown as an arrow connecting these groups.

HGT is a major force shaping genome evolution in bacteria (18, 19). To evaluate whether HGT may have contributed to the evolution of the Feo system, we constructed a phylogenetic tree from the concatenated sequences of all Feo proteins encoded in each locus. As shown in Fig. 3, Feo sequences clustered mainly according to their phylogenetic relationship, agreeing with the organismal phylogeny shown in Fig. 2. Yet the sequences from some species group within unrelated clades, which may indicate HGT events involving the *feo* genes. Namely, *Xanthomonas* and *Xylella* species, both *Gammaproteobacteria*, localize within the *Betaproteobacteria* group, with bootstrap support of 99%. These plant pathogens likely acquired *feoABC* from *Betaproteobacteria* via HGT, possibly from other pathogens that occupy the same niche, such as *Ralstonia* species (20). Interestingly, the *feo* genes of the latter species are often carried on megaplasmids, which could facilitate transfer events involving these genes (see Table S5 at https://doi.org/10.6084/m9.figshare.17082758). This association remains when analyzing FeoA and FeoC sequences separately (see Fig. S2 and S3 at https://doi.org/10.6084/m9.figshare.17082773), indicating that this potential HGT event moved the whole Feo system rather than independent genes. Overall, the FeoA tree largely recapitulates the same associations as those in the FeoC tree, suggesting that FeoA and FeoC have evolved together; thus, there is no evidence suggesting that *feoC* was acquired independently in other species via HGT.

### Phylogenetic reconstruction of the Feo system reveals a complex evolutionary history.

In our phylogenetic tree of the Feo system (Fig. 3), we included all the Feo sequences annotated in the analyzed genomes. Some species have several Feo systems with up to two *feoA* genes. Besides HGT events and mutations that might have led to dispensing with FeoC, our tree shows evidence of a complex evolutionary history. For instance, some Feo systems encoded in the same genome do not cluster together in our tree. Rather, they are grouped into clades with Feo systems from different species. This is the case for Feo systems found in *Firmicutes*, *Cyanobacteria*, and *Alphaproteobacteria*. Although these phyla form monophyletic groups in the organismal tree (Fig. 2), they are paraphyletic groups in the Feo tree. The multiplicity of *feo* genes in these species could indicate that they resulted from an evolutionary history that includes gene duplication events followed by subfunctionalization.

An instance of this more complex evolutionary history occurs in Porphyromonas gingivalis W83. This species contains two Feo operons, one of which has been demonstrated to be an Mn^2+^ transporter (21, 22). In our tree, this system forms a monophyletic group with 100% bootstrap support together with several Feo systems found in the genomes of *Firmicutes* species. This finding suggests that a family of Mn^2+^ transporters evolved from the Feo system due to gene duplication.

Another significant disagreement between the Feo and the organismal phylogenies is found in the alphaproteobacterial species, as they do not form a single monophyletic group in the Feo tree (Fig. 3). Rhodobacter capsulatus has two functional Feo operons (see Fig. S4 at https://doi.org/10.6084/m9.figshare.17082773), one of which has two *feoA* genes and an *feoC* gene and is conserved in Rhodobacter sphaeroides, while the other one, which is found only in Rhodobacter capsulatus, lacks *feoC* and likely functions as an Mn^2+^ transporter (labeled 2 in Fig. 3) (23). The R. capsulatus Feo operons do not cluster together in our tree, suggesting different evolutionary origins. Similarly, Magnetospirillum magneticum has two distinct Feo operons. FeoB1, which belongs to the more diverged operon in our phylogeny, is the main Fe^2+^ transporter and has an accessory role in magnetosome formation, as demonstrated in Magnetospirillum gryphiswaldense (24, 25). On the other hand, the operon containing FeoB2, which clusters together with other alphaproteobacterial Feo systems, is a minor contributor to iron uptake and magnetosome formation, but it is equally involved in the oxidative stress response (24, 25).

### Shewanella oneidensis FeoA and FeoB are necessary and sufficient to support iron uptake.

**FIG 4:**
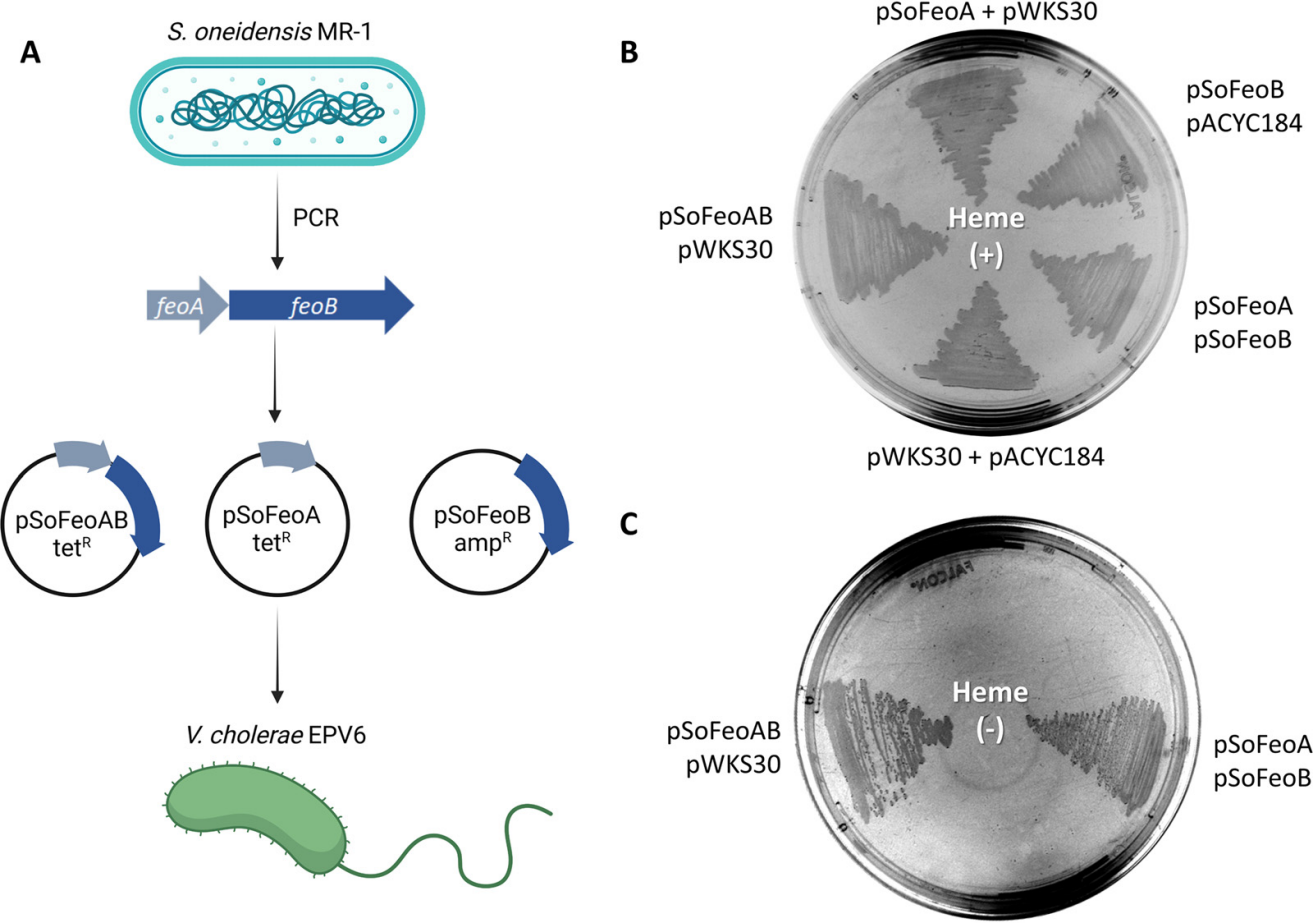
FeoA and FeoB from Shewanella oneidensis MR-1 (SoFeoA and SoFeoB) are necessary and sufficient to support iron uptake. (A) V. cholerae EPV6 cells were transformed simultaneously with different binary combinations of pSoFeoA, pSoFeoB, pSofeoAB, and the corresponding empty vectors (pACYC184 and pWKS30). (B) Control plate of LB agar supplemented with heme. V. cholerae EPV6 grows in this medium regardless of the presence or absence of a functional Feo system. (C) The same strains were plated onto nonsupplemented LB agar in the same order. Only the strains of V. cholerae EPV6 harboring both the *SofeoA* and *SofeoB* genes, either in a single vector or in separate vectors, grew in this medium.

We inferred from the *Shewanella* genomes that these species do not require FeoC. To validate this observation, we cloned the *feoAB* genes of S. oneidensis MR-1 (type strain) into the pACYC184 vector to determine whether these genes encode a functional Feo system on their own. V. cholerae EPV6 carrying this construct was able to grow in Luria-Bertani (LB) medium without heme supplementation, indicating that S. oneidensis *feoAB* encodes a functional Feo system and does not require a separate FeoC (Fig. 4). Also, by expressing these genes from separate vectors, we confirmed that neither FeoA nor FeoB alone supports iron uptake. Therefore, FeoA and FeoB from S. oneidensis are necessary and sufficient to assemble a functional iron transporter. We confirmed these results using E. coli H1771 as an alternative host to assess iron transport (see Fig. S5 at https://doi.org/10.6084/m9.figshare.17082773) (26).

### Cysteine residues in E. coli FeoC are required for full function.

**FIG 5:**
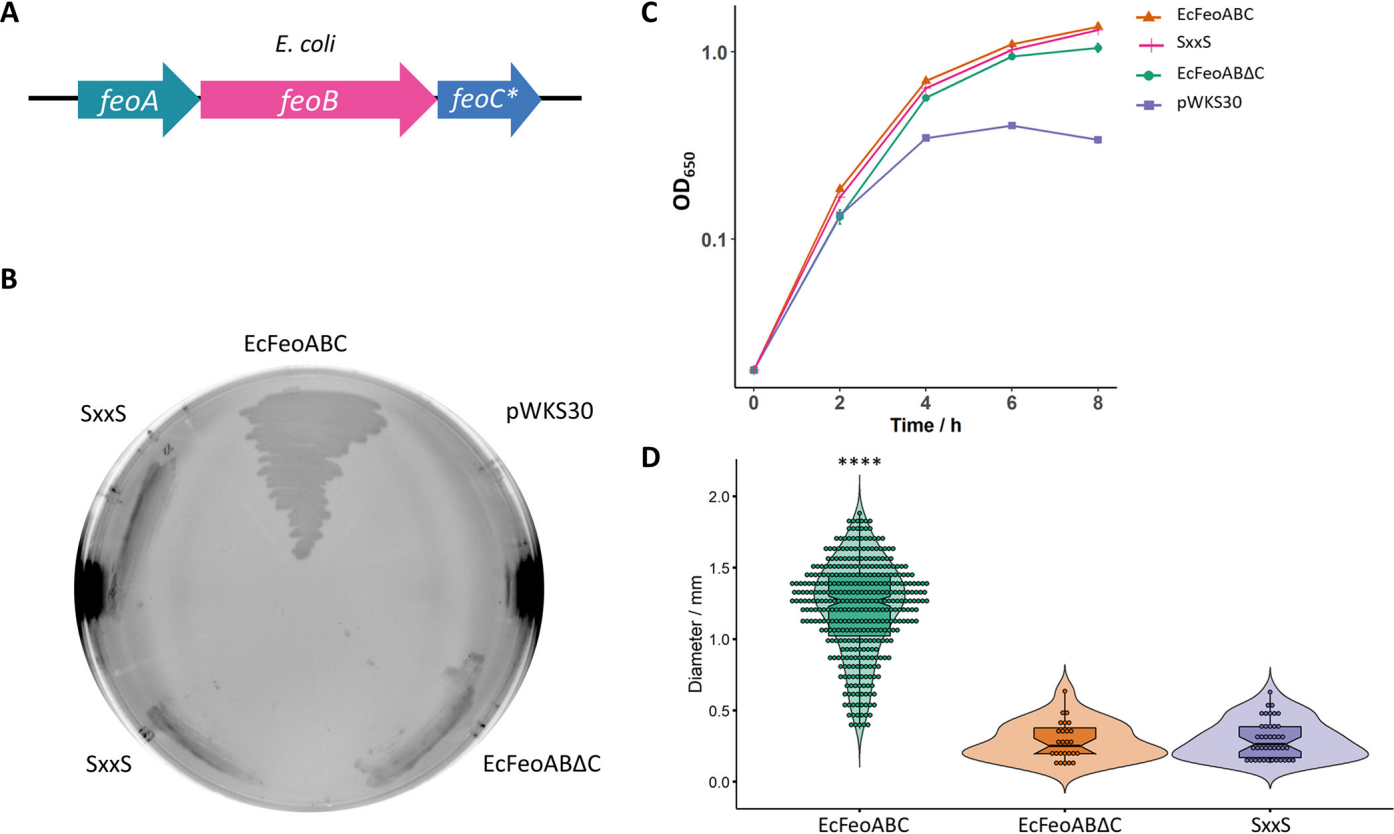
The mutation SxxS in the cysteine-rich motif of E. coli FeoC leads to a phenotype similar to that caused by the deletion of FeoC. (A) Schematic representation of the tested genetic constructs: E. coli *feoABC* (EcFeoABC) or mutants in FeoC (feoC*), namely, the FeoC C61S and C64S double mutant (SxxS) and the FeoC deletion mutant (EcFeoABΔC). (B) V. cholerae EPV6 grows on LB agar without heme supplementation when transformed with a plasmid harboring EcFeoABC. Conversely, the FeoC mutants do not support EPV6 growth in nonsupplemented medium. (C) In liquid medium, EcFeoABC as well as the two FeoC mutants support EPV6 growth. Only the strain carrying the empty vector (pWKS30) shows a growth defect under these conditions (*n* = 3). (D) The difference in growth rates in solid medium was quantified by measuring colony size after 24 h of growth on agar plates. The distributions of all the groups were compared by the Kruskal-Wallis test with Dunn’s multiple-comparison test. **** indicates a *P* value of <0.0001.

Since the most conserved feature of FeoC sequences is the cysteine-rich motif, we tested whether these residues are required for Feo-mediated iron transport. E. coli FeoC contains four cysteines arranged as a ^56^CX_4_CX_2_CX_5_C^70^ motif in proximity to its C terminus. We replaced the two central cysteines with serines, obtaining the ^61^SxxS^64^ mutant, and assessed the phenotype of V. cholerae EPV6 carrying a plasmid with this mutation. The strain harboring the FeoC^SxxS^ mutant showed a phenotype similar to that of the ΔFeoC mutant, indicating that the conserved C residues in E. coli FeoC are necessary for full function (Fig. 5). In contrast to V. cholerae Feo, the deletion of FeoC in the E. coli Feo system does not completely abolish growth in EPV6, particularly in liquid media (Fig. 5C).

### Evidence that *Vibrio* Feo systems may represent a transitional form on an evolutionary pathway to FeoC loss.

In our phylogenetic reconstruction of Feo, *Gammaproteobacteria* are a paraphyletic group in which *Vibrio*, *Aliivibrio*, and *Shewanella* species form a clade separate from the other species (Fig. 3). This clustering pattern is recapitulated when analyzing FeoA and FeoC sequences separately (see Fig. S2 and S3 at https://doi.org/10.6084/m9.figshare.17082773). Unlike most *Gammaproteobacteria*, the *Shewanellaceae*, *Alteromonadaceae*, and *Pseudoalteromonadaceae* species in the tree lack FeoC, but their closest neighbors within the *Alteromonadales* family (i.e., *Psychromonadaceae* and *Moritellaceae*) have FeoC. Moreover, unlike most species, including other *Vibrionaceae* (i.e., *Aliivibrio*, *Photobacterium*, *Grimontia*, *Salinivibrio*, and *Enterovibrio*), FeoC sequences found in *Vibrio* spp. have no cysteine residues (see Fig. S3 and S6 at the URL mentioned above); thus, they contain neither the CxxC nor the CxxGxCKxCPx_4–7_C archetypic motif. Taken together, these findings show that cysteineless FeoC is a characteristic feature of the *Vibrio* lineage, which likely evolved after the divergence of this genus within the *Vibrionaceae* family (27). This also suggests that the Feo components of *Vibrio* and some *Alteromonadales*, while required for Feo function, may play a different role than the FeoC found in other *Gammaproteobacteria*. Hence, the divergence of the FeoC elements in this group could reflect that they are similar to transitional forms such as those that may have existed on the evolutionary pathway from FeoC-dependent transporters (such as in *Vibrio*) to FeoC-independent transporters in other groups (such as in *Shewanella*).

**FIG 6:**
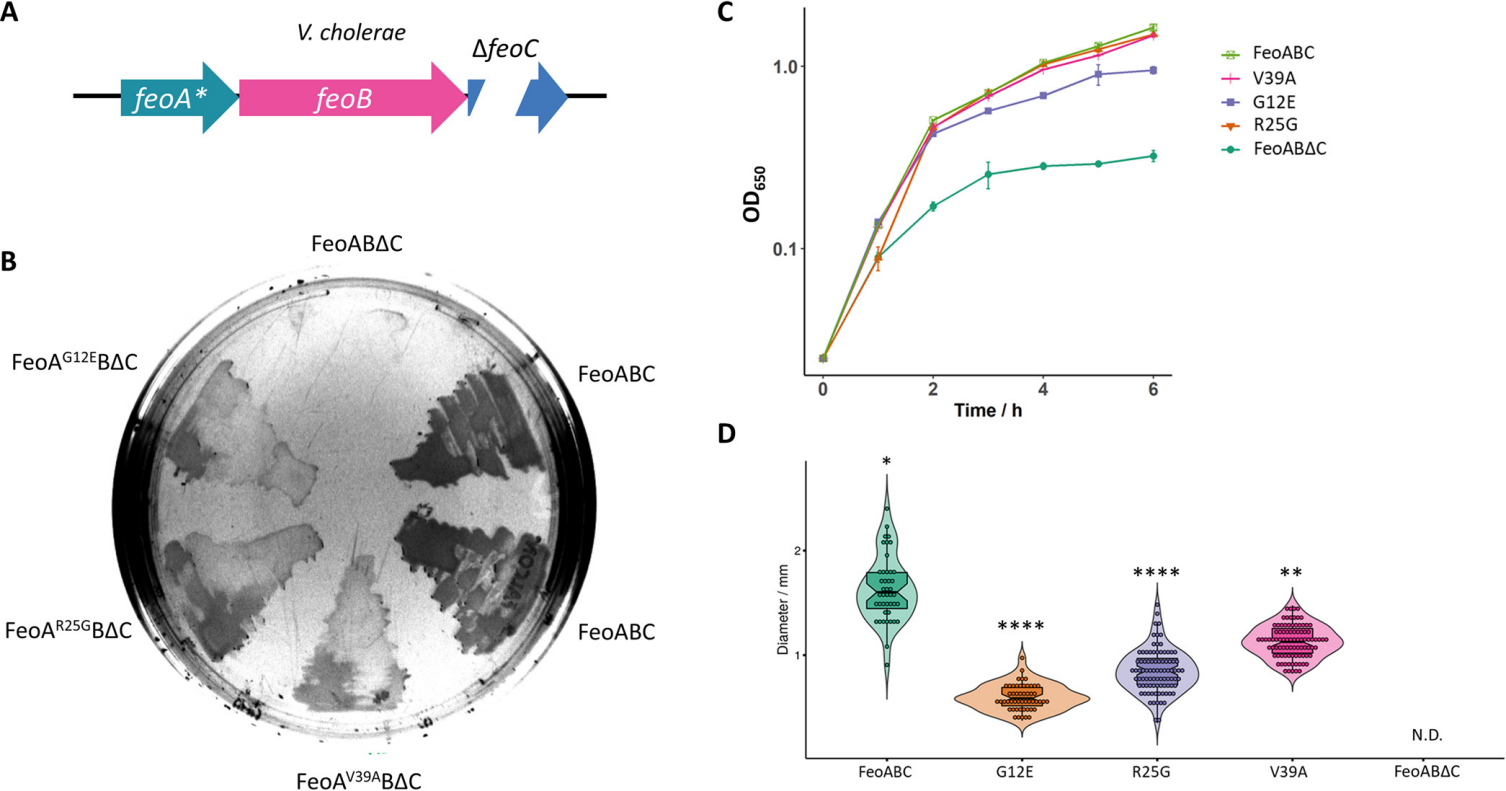
Single amino acid substitutions in FeoA partially bypass the requirement for FeoC in V. cholerae. (A) Schematic representation of the tested genetic constructs. The V. cholerae *feoAB*Δ*C* operon with a single substitution in *feoA* is indicated. (B) V. cholerae EPV6 grows on LB agar without heme supplementation when transformed with a plasmid harboring the wild-type FeoABC system. The same construct containing an in-frame deletion in *feoC* (FeoABΔC) does not support EPV6 growth in nonsupplemented medium. Cells transformed with the deletion mutant containing a single substitution in FeoA (G12E, R25G, or V39A) showed an intermediate growth phenotype on solid medium. (C and D) Similar results were obtained by assessing cell growth in LB broth (*n* = 3) (C) or by measuring colony size after 24 h of growth on agar plates (D). The strain carrying FeoABΔC did not develop visible colonies (not detected [N.D.]). The distribution of each group was compared to that of the control group (FeoABC) by the Kruskal-Wallis test with Dunn’s multiple-comparison test. ** and **** indicate *P* values of <0.01 and <0.0001, respectively.

If *Vibrio* Feo is at an intermediate stage between the FeoC-requiring ancestor and the FeoC-independent phenotype, then FeoA and FeoB from both *Shewanella* and *Vibrio* might share some of the mutations needed to bypass the requirement for FeoC. To determine whether spontaneous changes may render the V. cholerae Feo system independent of FeoC, we induced random mutations into the V. cholerae Feo operon containing an in-frame deletion of *feoC*. Next, we selected those mutants in which ferrous iron transport was rescued. Specifically, we transformed plasmids carrying the mutagenized operons into the iron transport-deficient V. cholerae strain EPV6, which grows only in medium supplemented with heme or when it harbors a functional Feo system (28). Through this approach, we isolated three colonies growing in nonsupplemented medium as presumptive FeoC-independent mutants. They all carried a single amino acid substitution in FeoA: G12E, R25G, or V39A. To test whether these mutations were responsible for the observed phenotype, we introduced them *de novo* into a clean Δ*feoC* background by site-directed mutagenesis. Each amino acid substitution in FeoA partially bypassed the requirement for FeoC in V. cholerae EPV6. Cells harboring any of these mutations survived and showed growth rates intermediate between that of the wild-type and that of the initial Δ*feoC* mutant (Fig. 6). Because Δ*feoC* is correlated with reduced levels of FeoB in V. cholerae (10) and S. enterica (14, 15), we tested the effect of the suppressor mutations on FeoB levels. If the primary function of FeoC is to stabilize FeoB, then suppressors that restore function should also restore wild-type levels of FeoB. However, we did not observe increased levels of FeoB or Feo complexes in the suppressors (see Fig. S7 at the URL mentioned above). Therefore, single mutations in FeoA can, at least partially, bypass the requirement for FeoC, and the restoration of Feo function is not due to increasing the amount of FeoB.

**FIG 7:**
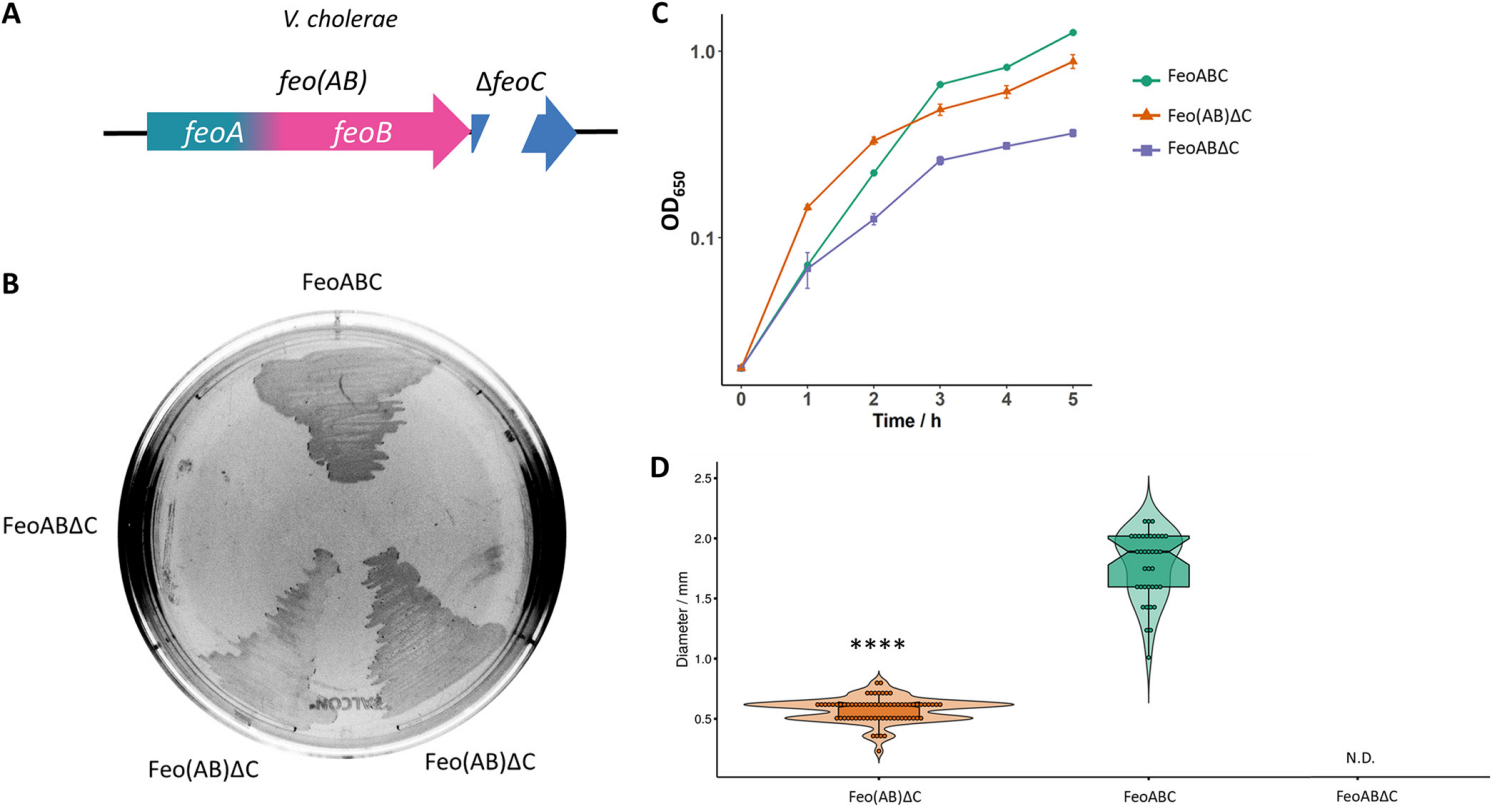
The Feo(AB) fusion partially bypasses the requirement for FeoC in V. cholerae. (A) Schematic representation of the tested genetic constructs. V. cholerae *feoA* and *feoB* were fused in a single ORF in an *feoC* deletion background to generate Feo(AB)ΔC. (B) V. cholerae EPV6 grows on LB agar without heme supplementation when transformed with a plasmid harboring the wild-type FeoABC system. The same construct containing an in-frame deletion in *feoC* (FeoABΔC) does not support cell growth in nonsupplemented medium. Cells transformed with a variant of Feo(AB)ΔC exhibit intermediate growth in solid medium. (C and D) Similar results were obtained by assessing cell growth in LB broth (*n* = 3) (C) or by measuring colony size after 24 h of growth on agar plates (D). The strain carrying FeoABΔC did not develop visible colonies (N.D.). The distributions of both groups were compared by the Mann-Whitney test. **** indicates a *P* value of <0.0001.

Since several Feo systems lacking FeoC exhibit an FeoA-like domain at the N terminus of FeoB (Fig. 1), we also assessed the phenotype associated with a fusion between *feoA* and *feoB* in V. cholerae. Strikingly, the fusion protein, referred to as Feo(AB), partially bypassed the requirement for FeoC as well (Fig. 7). However, combining the Feo(AB) fusion with any of the single substitutions in FeoA described above did not further enhance the growth of V. cholerae EPV6 (see Fig. S8 at the URL mentioned above), indicating that different additional mutations are needed to fully recapitulate the wild-type phenotype in the Δ*feoC* background. We also evaluated whether the Feo(AB) fusion led to an FeoC-independent phenotype in other species by constructing such a fusion in the E. coli Feo system. However, the Feo(AB) fusion was insufficient to render the E. coli Feo system independent of FeoC (see Fig. S9 at the URL mentioned above). Similarly, we were unable to isolate FeoC-independent mutants for Δ*feoC* in the E. coli Feo system. Together, these results support the hypothesis that V. cholerae has already accumulated some of the mutations that might have led to FeoC independence in *Shewanella* so that only a few additional changes in FeoA and FeoB are needed to replace the diminished role of FeoC.

## DISCUSSION

Studies of the Feo system have largely focused on FeoB, the central functional and structural element of the system. However, Feo is a multicomponent transporter; thus, the roles and evolutionary histories of the accessory proteins FeoA and FeoC must be studied to gain a deeper understanding of this system. FeoA is a small cytoplasmic protein essential for Feo-mediated iron transport. It interacts with FeoB and is required for the assembly of the membrane pore in V. cholerae (10, 11). FeoC is also a small cytoplasmic protein, but its role has been particularly elusive (1). Although FeoC is essential for Feo function in some of the systems in which it is present, as shown in V. cholerae (11) and Salmonella enterica (14, 15), it is not widespread among species, and its sequence is poorly conserved.

The phylogenetic distribution of FeoC that we found recapitulates that reported in previous studies (2, 13), showing that this element is prevalent in *Gammaproteobacteria*, although other FeoC-like elements exist in other groups. Numerous hypotheses have been proposed regarding the function of FeoC, yet there is not enough evidence to fully validate or rule out any of them, and it appears that FeoC may have different roles in different species.

By performing a comparative assessment of Feo gene architecture in bacteria, we propose that FeoC was likely present in the common ancestor of several Feo systems but was subsequently lost in multiple lineages. If this model is correct, species lacking FeoC will have compensated for its function through changes in other elements, likely FeoA and FeoB. Since the presence or absence of FeoC occurs in many separate branches of the Feo tree, we propose that different lineages have dispensed with *feoC* through different changes in the Feo system.

Notably, while we propose gene loss as the most likely force shaping the current distribution of *feoC* in bacteria, our model is not inconsistent with some instances of *feoC* acquisition via HGT, as seen in *Xanthomonas* and *Ralstonia* species. Likewise, if FeoC-like proteins comprise more than one family of analogs or ancient orthologs, these families may have evolved through different mechanisms, including the acquisition of FeoC by the ancestor of the *Gammaproteobacteria* group followed by discrete loss events, such as that of *Shewanella* species. Therefore, the discussion on how the architecture of the Feo system evolved is still open. To evaluate the feasibility of different evolutionary models, establishing the actual relationship between the FeoC-like proteins is a priority.

We used the Feo systems of S. oneidensis and V. cholerae to investigate this hypothesis. Our phylogenetic analyses suggested that *Vibrio* species might already harbor an Feo genetic background prone to dispensing with FeoC, needing only subtle changes to evolve FeoC independence. Our experiments supported this model. We show that specific mutations or an Feo(AB) fusion allowed V. cholerae to obtain iron via Feo in the absence of FeoC. For comparison, the fusion of FeoA and FeoB did not bypass the requirement for FeoC in E. coli. This fusion requires the deletion of 19 nucleotides in E. coli (see Fig. S10 at https://doi.org/10.6084/m9.figshare.17082773) but only a single nucleotide insertion in V. cholerae (because *feoA* and *feoB* overlap one another). Thus, the Feo system of V. cholerae may be more likely to naturally acquire a mutation that results in an Feo(AB) fusion and eventually evolve to dispense with FeoC. We have previously shown that this fusion protein from V. cholerae is stable and active (12).

Because gene fusion/fission is the major factor governing the evolution of multidomain proteins (29), we propose that this type of mutation might have contributed to the evolution of the Feo system more generally. It has been shown that other multidomain proteins originating from gene fusions are positively selected and dispersed by HGT (30). Some species in the *Bacteroides* and *Firmicutes* groups may be further examples of this evolutionary pathway at work, as they have Feo(AB) fusion proteins and lack FeoC.

To gain insight into the function and mechanism of action of FeoC, we examined in more detail how its sequence evolved in the *Vibrionaceae* family. Previous studies suggest that this family of bacteria evolved from a common ancestor that existed about 600 million years ago in the Devonian, from which the genera *Vibrio* and *Aliivibrio* diverged (27). We noticed that most gammaproteobacterial FeoC sequences include a motif with four cysteines, while *Photobacterium* and *Aliivibrio* FeoC sequences have three cysteines, and *Vibrio* FeoC has none (see Fig. S6 at the URL mentioned above). Some authors have proposed that the widespread cysteine-rich motif in FeoC must have functional significance (1, 2). In E. coli FeoC, this cysteine-rich motif forms a [4Fe-4S] cluster under anaerobic conditions (16). Thus, it has been suggested that FeoC may function as a transcription factor responsive to iron.

However, *Vibrio* FeoC, which lacks cysteines, does not fit this model. Furthermore, the deletion of *feoC* did not significantly affect the expression of *feoB* in V. cholerae (11) or Yersinia pestis (31). In Salmonella enterica, FeoC protects FeoB from proteolysis (14, 15), but this cannot be the only role of FeoC. In V. cholerae, the loss of FeoC also results in reduced levels of FeoB protein, but increasing the expression of *feoB* in the absence of *feoC* did not restore iron transport, even though it restored wild-type levels of FeoB (10). We have shown that V. cholerae FeoC does not affect the catalytic activity of FeoB *in vitro* (12), indicating that the regulation of nucleoside triphosphate (NTP) hydrolysis is not the role of this protein either. We postulate that FeoC is likely to couple NTP hydrolysis with pore opening (12) so that those mutations that dispense with FeoC might cause structural changes to facilitate interactions with FeoA or between FeoB domains that are required for pore opening. Additional roles for FeoC are possible; indeed, it may have multiple functions that differ from one species to another.

It has been shown that the E29 and M35 residues in V. cholerae FeoC are essential for interacting with FeoB and for Feo-mediated iron transport in this species (11). Our analyses of the FeoC sequences across the *Vibrionaceae* family revealed that other amino acids are highly conserved within this group (see Fig. S6 at the URL mentioned above). Therefore, besides the role of the cysteine-rich motif, future studies could investigate whether other motifs have evolved to have critical functional significance in certain bacterial lineages.

HGT often shapes the evolution of gene clusters (18, 19). We found clear evidence of HGT involving the Feo operon between gamma- and betaproteobacterial plant pathogens. Specifically, it appears that the common ancestor of *Xanthomonas* and *Xylella* species acquired the Feo operon from *Betaproteobacteria*, and it was then fixed by positive selection. Instances of HGT between these plant pathogens have been documented, especially involving genes relevant for virulence (32). We infer that the betaproteobacterial Feo system, which contains a small cysteine-rich oligopeptide as an FeoC-like element, most likely provides an adaptative advantage for the pathogenic lifestyle of these species. Together with the high prevalence of FeoC in *Gammaproteobacteria*, this scenario is in contrast to our observation of FeoC independence in *Vibrio* and *Shewanella* species. This suggests that the maintenance of the *feoC* gene may be favored under certain conditions.

Whether putative FeoC elements, FeoB-associated cysteine-rich proteins, and cysteine-rich C-terminal FeoB domains are orthologs or paralogs is unclear. The FeoB-associated cysteine-rich proteins are putative open reading frames (ORFs) for small peptides prevalent in *Firmicutes* and *Bacteroidetes* (see Fig. S1 at the URL mentioned above), but whether they are translated into functional peptides is still an unanswered question. Also, cysteine-rich motifs are a common feature in most FeoC-like elements. Still, this feature could have arisen multiple times and may reflect convergent evolution for metal-binding or redox-sensing functions. Equally possible is that these alternative FeoC-like elements reflect the transition from systems with FeoC to systems lacking this component. In this regard, *Vibrio* species seem to be the exception that proves the rule insofar as this genus has an FeoC protein that is required for iron uptake but lacks cysteine residues. Overall, the role of FeoC remains elusive, but we propose that it may be coupling nucleotide hydrolysis with pore opening (12). This activity may not be its only role in *Vibrio* species, and FeoC-like elements could have different or additional functions in other species.

Bacterial metal transporters often have promiscuous specificity toward isovalent cations. Most importantly, there is an interplay between Fe^2+^ and Mn^2+^ in bacterial redox homeostasis (33), and transporters like the enterobacterial Sit/Yfe systems have dual activity toward both metals (34). In this vein, it is worth noting that some putative Feo systems likely encompass a homologous family of Mn^2+^ transporters, proposed as the “Meo system” (13). Namely, Porphyromonas gingivalis, a *Bacteroidetes* species, has two putative Feo operons, one of which is a proven Mn^2+^ transporter (21, 22, 25). R. capsulatus and M. gryphiswaldense also have two putative Feo systems that contribute differently to iron transport (23, 24). In the latter case, one of these systems has an accessory role in magnetosome formation and is involved in the oxidative stress response. Meo may have evolved from Feo by gene duplication (13, 23). In R. capsulatus, the operon potentially involved in Mn^2+^ transport lacks *feoC*. On the other hand, the operon dedicated to Fe^2+^ transport, which is also conserved in R. sphaeroides, has an FeoC-like element and is in a region enriched in iron uptake genes such as siderophore synthases and transporters (see Fig. S4 at the URL mentioned above).

*Firmicutes* display a remarkable multiplicity and diversity of Feo proteins, having up to three operons in a single genome, some with several *feoA* genes. In our Feo tree, some Feo clusters from *Firmicutes* form a monophyletic group with the P. gingivalis Mn^2+^ transporter but separate from that of R. capsulatus mentioned above. If these systems are true Mn^2+^ transporters, these results indicate that specialization toward Mn^2+^ may have resulted from gene duplication events that occurred in several lineages. Alternatively, an ancestral Mn^2+^ transporter derived from Feo may have been horizontally transferred between these groups, although they do not cluster together as a monophyletic group in our Feo tree. Further research is needed to evaluate whether these systems can support the transport of metals other than Fe^2+^. The functional significance of multiple FeoA proteins in a single operon also merits further study.

Finally, an unexpected result of our search was finding putative Feo genes in *Archaea* and Neocallimastigomycota genomes (see Tables S1 and S2 at https://doi.org/10.6084/m9.figshare.17082758). The latter group is the earliest-diverging fungal phylum, which comprises anaerobic unicellular symbionts that inhabit the gastrointestinal tract of large mammalian herbivores (35), an anoxic and acidic environment where Fe^2+^ is likely the primary source of iron. Hantke (36) proposed that the Feo system may be a “living fossil” of the eukaryotic G proteins. Thus, Feo genes in eukaryotes might represent a key piece of evidence for evolutionary studies. Yet the presence and functionality of Feo homologs in these fungi still need to be experimentally validated. If these fungal systems are homologs, they may have been either preserved through evolution or horizontally acquired from bacteria, as HGT between these groups is not uncommon (35, 37, 38). Hence, Feo genes found in eukaryotes merit further investigation.

### Conclusions.

**FIG 8:**
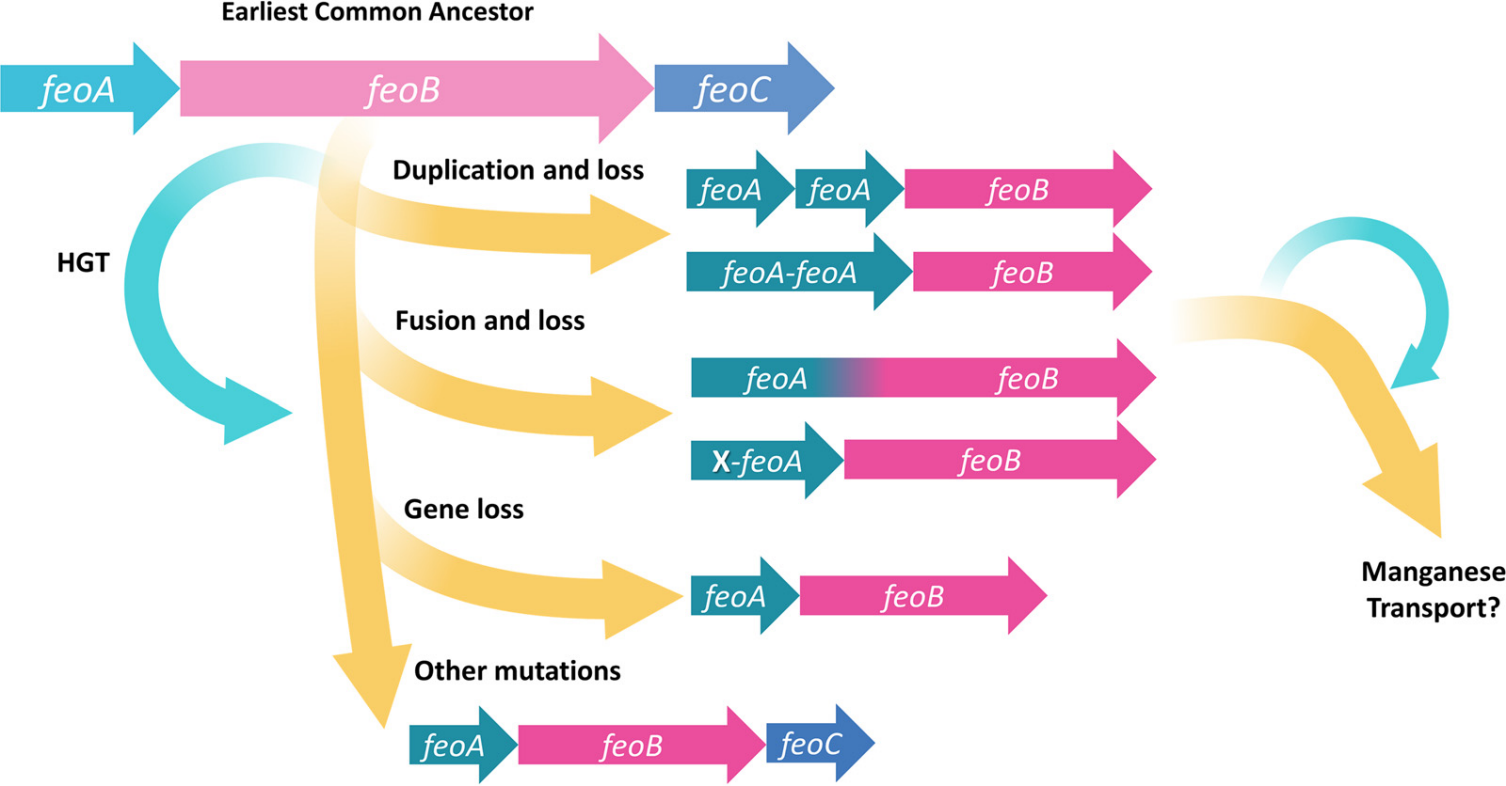
Proposed evolutionary model of the Feo system. We hypothesize that the earliest common ancestor of the Feo system had all three Feo elements: FeoA, FeoB, and FeoC. Through evolutionary changes, including gene duplications, gene fusions, and horizontal gene transfer (HGT), most lineages dispensed with FeoC through compensatory changes in FeoA and/or FeoB. Only a few groups still maintain FeoC. Some Feo systems might have acquired specificity toward the isovalent cation Mn^2+^ and could comprise a family of “Meo” transporters. We present experimental data consistent with this model in *Gammaproteobacteria*, but further characterization of Feo proteins in other groups is needed.

In this study, we analyzed the evolutionary history of Feo, the major system for ferrous iron transport in bacteria. We evaluated how HGT, gene duplication, gene fusion, and gene loss have shaped the evolution of the Feo system. We hypothesize that Feo emerged as a three-component system before the divergence of the main bacterial phyla and then evolved differently in different lineages, acquiring a broad spectrum of architectures and even giving birth to other transport systems. The evolutionary model that we propose is summarized in Fig. 8. Although we tested possible evolutionary pathways to dispense with FeoC in Vibrio cholerae, a generalized lack of experimental evidence is still a major gap toward a complete evolutionary and mechanistic understanding of Feo. Therefore, future research must prioritize the elucidation of the roles of FeoA and FeoC as well as the characterization of the Feo system in different species.

## MATERIALS AND METHODS

### Identification of *feo* genes in bacterial genomes.

To identify Feo gene clusters annotated in bacterial genomes as a function of FeoA and FeoC, the HMM profiles of these proteins deposited in Pfam (PF04023 and PF09012 for FeoA and FeoC, respectively) were used as queries to search in the UniProt Reference Proteomes database through HMMER (17), specifically by using the HMMSEARCH program. Those genomes containing matches for FeoA and/or FeoC were used as a database in HMMERSEARCH to identify the presence of NFeoB (PF02421). The results from all searches were cross-checked to determine the gene architecture of each locus, including the presence, multiplicity, and location of *feoA*, *feoB*, and *feoC*.

Results from fungal genomes were manually inspected to rule out spurious sequences. With this aim, BLAST analysis of the amino acid sequences under inspection was performed (by BLASTP) against the nonredundant protein sequence database (NCBI). Those sequences with significant hits in genomes other than those retrieved in the original search (e.g., annotated in bacteria) were considered potential contaminants or annotation errors and were not used in further analyses. Those results that passed this curation are shown in Table S1 at https://doi.org/10.6084/m9.figshare.17082758.

### Phylogenetic reconstructions.

Amino acid sequences (see Table S6 at the URL mentioned above) were aligned using MUSCLE through MEGAX software (39), with all settings defined by default. The resulting alignments were then used to construct the corresponding phylogenetic trees by the maximum likelihood method with the Jones-Taylor-Thornton model and the gamma distribution for evolutionary rates. Cutoff values were fixed at 95% for coverage, and trees were tested by bootstrapping with 300 replicates.

For the Feo system tree, the amino acid sequences of all the Feo proteins encoded in the same locus were concatenated and then analyzed as described above. Of note, *feo* genes in *Helicobacter* species are not adjacent to one another, but since no other copies were detected in *Helicobacter* genomes, they were concatenated as a single sequence to construct this tree.

To check that no *feoC* genes were overlooked because they were not annotated as reading frames, those genomes with no *feoC* were analyzed by TBLASTN against the FeoC-like sequences of the closest species. Additionally, for those Feo clusters lacking *feoC*, the 50 last 3′ bp of *feoB* together with the next downstream 450 bp were analyzed using ORF Finder (https://www.ncbi.nlm.nih.gov/orffinder/), and any potential ORF longer than 40 aa was used as a query in BLASTP against the nonredundant protein sequence database.

### Reagents, bacterial strains, and growth conditions.

All chemicals and growth media were purchased from Sigma-Aldrich Chemical Company. Bacterial strains were preserved at −80°C in tryptic soy broth (TSB) with 20% glycerol. Unless otherwise stated, E. coli and V. cholerae strains were grown in Luria-Bertani (LB) broth (10 g L^−1^ tryptone, 5 g L^−1^ yeast extract, and 10 g L^−1^ NaCl in double-distilled water) or on LB agar (1.5% [wt/vol] bacteriological agar) at 37°C and at 200 rpm for liquid media.

Bacterial strains and plasmids used in this study are described in Tables S7 and S8, respectively, at https://doi.org/10.6084/m9.figshare.17082758. For strains harboring plasmids, the following antibiotics were used: 50 μg mL^−1^ ampicillin, 50 μg mL^−1^ kanamycin, and 12.5 μg mL^−1^ tetracycline for E. coli and 25 μg mL^−1^ ampicillin, 6.25 μg mL^−1^ tetracycline, and 5.0 μg mL^−1^ gentamicin for V. cholerae. When indicated, heme was added at 10 μM.

### Site-directed mutagenesis.

Primers used in this study are listed in Table S9 at https://doi.org/10.6084/m9.figshare.17082758. Point mutations in V. cholerae were generated in the corresponding parental plasmids by the QuikChange protocol (40) for site-directed mutagenesis, using the primers FeoA-N-F and FeoA-N-R (where N refers to the targeted change, either G12E, R25E, or V39A). Similarly, the *feo*(*AB*) fusion in the V. cholerae operon was generated through site-directed mutagenesis with the primers Vcfeo(AB)fus-F and Vcfeo(AB)fus-R. For E. coli *feo* genes, the *feo*(*AB*) fusion was generated in pEcFeoAB by PCR-mediated deletion (41) with the primers Ecfeo(AB)fus-F and Ecfeo(AB)fus-R. pEcFeoABC^SxxS^ was generated by inserting the C61S and C64S substitutions into the *feoC* gene in pEcFeoABC using the primers EcFeoC-SxxS-F and EcFeoC-SxxS-R.

### Cloning of the S. oneidensis *feo* genes.

Genomic DNA from S. oneidensis MR-1 was kindly donated by Benjamin Keitz (Department of Chemical Engineering at The University of Texas at Austin). Primer pair SoFeoA-EcoRI-F and SoFeoA-EcoRI-R and primer pair SoFeoA-EcoRI-F and SoFeoB-NotI-R were used to amplify S. oneidensis *feoA* (*SofeoA*) and *SofeoAB*, respectively. The PCR product for *SofeoA* was digested with EcoRI and cloned into the corresponding restriction site in pACYC184 in the same direction as the P*cam*^r^ promoter. The PCR product for *SofeoAB* was digested with EcoRI and NotI and cloned into the corresponding restriction sites in pWKS30 in the same direction as the P*lac* promoter. To generate pSoFeoB, *SofeoAB* was amplified with SoFeoA-EcoRI-F and SoFeoB-EcoRI-R and cloned into pACYC184 in the same direction as the P*cam*^r^ promoter. Next, *SofeoA* was removed by PCR-mediated deletion using the primers SoFeoA-del-F and SoFeoA-delR. PCRs were done using high-fidelity Phusion *Taq* polymerase (New England BioLabs). The sequence and directionality of all the constructs were verified by Sanger sequencing at the DNA Sequencing Facility at The University of Texas at Austin.

### Growth assessment of V. cholerae EPV6.

V. cholerae EPV6 (O395 Δ*vibB feoABC*::*kan vctP*::*gent fbpA*::*cam*) (28) cells carrying the Feo constructs under analysis were streaked onto different quadrants of LB agar plates, with and without heme supplementation, and incubated at 37°C overnight. In these assays, functional Feo systems and empty vectors were used as positive and negative controls, respectively. For replication, all these assays were carried out in three separate plates under each condition; each plate was inoculated with individual colonies. Observable growth after incubation was considered a positive result.

Growth rates in liquid medium were measured as the optical density at 650 nm (OD_650_) in triplicate cultures in EZ rich defined medium (RDM) broth (http://www.genome.wisc.edu/functional/protocols.htm) supplemented with 2% (wt/vol) sucrose, 5 μM FeSO_4_, and the appropriate antibiotics. The absorbance was measured every hour for 6 h.

### Colony size assay.

A single colony of V. cholerae EPV6 harboring the Feo construct under evaluation was used to inoculate 5 mL of LB broth with the appropriate antibiotics and grown overnight at 37°C in an orbital shaker. Next, 10^−7^ and 10^−8^ dilutions of this culture were plated in triplicate onto LB agar supplemented with 10 μM FeSO_4_ and the corresponding antibiotics and incubated for 24 h at 37°C. Afterward, the plates were photographed, and the area of every colony was estimated from the photographs using ImageJ software (42). The colony diameter was calculated from the colony area assuming circularity.

### Assessment of iron transport in E. coli H1771.

Iron transport by different Feo constructs was evaluated in E. coli H1771 according to the protocol described previously by Marlovits and colleagues (43). In short, single colonies of E. coli H1771 transformed with the Feo system under evaluation were streaked separately onto LB agar supplemented with 150 μM FeSO_4_, isopropyl-β-d-thiogalactopyranoside (IPTG), 5-bromo-4-chloro-3-indolyl-β-d-galactopyranoside (X-gal), and the appropriate antibiotics. In this assay, β-galactosidase activity negatively correlates with iron transport activity; therefore, white colonies are read as a positive result, while blue colonies are considered a negative result.

### Isolation of FeoC-independent mutants.

The *feoAB*Δ*C* operons from E. coli or V. cholerae were cloned into pWMB1 (a mobile derivative of pWKS30 with *oriT*). E. coli BMH71-18mutL was transformed with either of these constructs. Next, one transformant colony was picked and grown overnight in 5 mL of LB broth to be used as a donor strain in a triparental mating with V. cholerae EPV6 (recipient) and E. coli MM294 carrying pRK2013 (helper). Cells from 1 mL of a mid-log-phase culture of each strain were pelleted and washed three times with saline to remove traces of antibiotics. Afterward, the cells were resuspended in 100 μL of fresh LB broth, and a mixture containing 25 μL of each resuspension was spotted onto an LB agar plate and incubated upside up at 37°C overnight. The cells were then scraped into 1.0 mL LB broth and plated onto LB agar plates supplemented with ampicillin, kanamycin, and gentamicin to recover V. cholerae EPV6 colonies.

Plasmids from the presumptive *feoC*-independent mutants were isolated and sequenced to identify mutations in the *feo* genes. These mutations were then inserted *de novo* into the initial construct (pFeoΔC) by site-directed mutagenesis to be tested in V. cholerae EPV6.

### Statistical analysis and graphs.

Average colony sizes from different Feo constructs were compared to that of the control group (FeoABC) by the one-way Kruskal-Wallis test with Dunn’s test for multiple comparisons. For 2-group comparisons, the Mann-Whitney test was used. These analyses and the corresponding graphs were made using R statistical software version 4.1.0 (44) and the ggplot2 package (45). Sequence logo plots were made by using Skylign software (46).

### Data availability.

No new sequences were generated in support of this study. All the sequences and HMM profiles underlying this article are available in the NCBI, Pfam, or UniProt public databases; their corresponding accession numbers and other data supporting this study are available within the document and in its online supplemental material. Supplemental material for this article is publicly available at Figshare. Supplemental tables can be found at https://doi.org/10.6084/m9.figshare.17082758, and supplemental figures can be found at https://doi.org/10.6084/m9.figshare.17082773.
